# Phage Therapy: Concept to Cure

**DOI:** 10.3389/fmicb.2012.00238

**Published:** 2012-07-19

**Authors:** Eric C. Keen

**Affiliations:** ^1^Department of Natural Sciences, Montgomery CollegeGermantown, MD, USA

The development and mass-production of antibiotics ranks as one of the twentieth century's greatest scientific achievements. For more than 60 years, antibiotics have comprised Western medicine's primary defense against bacterial disease. But although antibiotics have saved millions of lives, our chemical shield has become increasingly leaky. Just last year, the director-general of the World Health Organization warned that “the world is on the brink of losing these miracle cures [antibiotics]” and that “in the absence of urgent corrective and protective actions, the world is heading toward a post-antibiotic era, in which many common infections will no longer have a cure and, once again, kill unabated” (Liljeqvist et al., 2012).

Unfortunately, game-changing help from new antibiotics does not appear to be forthcoming. Over the past 30 years, the number of antibiotics newly approved in the United States has steadily declined, and despite increased awareness and redoubled efforts, the current R&D pipeline remains largely dry (Hughes, 2011). Even when effective antibiotics are available, it is becoming increasingly apparent that broad-spectrum antibiotics can have sustained and detrimental effects on the body's communities of beneficial bacteria (Buffie et al., 2011) which, according to a growing body of research, play a vital role in human nutrition (Yan and Polk, 2004) and immunity (O’Hara and Shanahan, 2006).

Given the underlying economic factors that make antibiotic development unprofitable, (Nathan and Goldberg, 2005) and given that abuses of antibiotics continue to drive bacterial resistance, it seems unrealistic to assume that antibiotics alone will prove sufficient to counter the long-term medical threat posed by drug-resistant bacteria. Rather than continuing to focus solely on chemical solutions to drug-resistance, which are ultimately static responses to a dynamic system, we must also seek approaches that can keep pace with the bacteria they are designed to kill. This idea is not futuristic or even theoretical: we can tap into an ancient arms race between bacteria and their viral predators, bacteriophages, to combat drug-resistant bacteria. Phage therapy, or the use of bacteriophages to kill pathogenic bacteria, represents a potentially significant, if currently underdeveloped, weapon in our ongoing battle against bacterial disease.

The purpose of this piece is not to provide a comprehensive history of phage therapy, but in order to understand its future prospects in Western medicine, some basic history is helpful. Bacteriophages, or “bacteria eaters” in Latin, were independently discovered by two microbiologists, Frederick Twort and Felix d'Herelle, in the late 1910s (Lederberg, 1996). Almost immediately, d'Herelle understood that these natural antagonists of bacteria represented a powerful new way of treating bacterial infections. The breakthrough came in 1919, when d'Herelle used phages to cure four patients of dysentery, and from there, phage therapy, when conducted by knowledgeable scientists like d'Herelle, met with significant success. D'Herelle used phages to halt outbreaks of cholera in India and plague in Egypt, and in 1923, two physicians from Baylor University's College of Medicine, reporting successful results from one of the first phage therapy trials conducted in the United States, concluded that “the bacteriophage holds enormous possibilities as a new weapon for fighting infectious disease” (Ho, 2001).

Despite (or perhaps because of) the heady enthusiasm for phage therapy, clinical results often failed to match the hype. Although phage products soon became commercially available in the United States, Western Europe, and the nascent Soviet Union, not every practitioner possessed d'Herelle's expertise, and largely because of a poor understanding of basic phage biology, early returns from phage therapy were mixed. In 1932, one American health officer presciently warned, “Because of conflicting experimental observations, enthusiastic and poorly controlled clinical application and rapidly expanding commercial exploitation, a situation is developing which will, unless guided and checked, lead to the ultimate rejection of bacteriophage by all who make any pretense to the practice of scientific medicine” (Larckum, 1932).

This rejection of phage therapy, which began with the misuse of phages, became complete with the emergence of antibiotics. Initially, antibiotics were cheap, widely available, and extremely effective against nearly all bacterial diseases, and because this golden age of antibiotic infallibility seemed like medicine's new *status quo*, phage therapy was discarded throughout the West as an unnecessary approach to an already-solved problem.

As has become uncomfortably apparent, though, bacterial diseases are not a solved problem, and scientists are now looking back to phages as a way to treat the most intractable bacterial infections. In the process, it has become clear that phage therapy holds some important advantages over traditional chemotherapy. Most importantly, phages are extremely precise: any given phage only attacks a very particular strain of bacteria. In d'Herelle's time, this fact often confounded, rather than aided, effective treatment, but today, advances in diagnostic technologies, such as real-time PCR (Espy et al., 2006), 16s rRNA sequencing (Rhoads et al., 2012), and laser-induced breakdown spectroscopy (Mohaidat et al., 2012), seem likely to greatly facilitate the rapid selection of appropriate phages. From a therapeutic perspective, this means that phage therapy can eliminate an individual patient's infection without affecting the body's communities of beneficial bacteria. In this light, phage therapy represents a kind of biomedical “smart bomb,” affecting only a specific target and minimizing collateral damage. Indeed, side effects from phages in human studies involving both sick (Rhoads et al., 2009) and healthy (Bruttin and Brussow, 2005) individuals have been extremely rare.

Moreover, although bacteria can become resistant to phages, phage-resistance is not nearly as worrisome as drug-resistance. Like bacteria but unlike antibiotics, phages mutate and therefore can evolve to counter phage-resistant bacteria (Matsuzaki et al., 2005). Because phages attack bacteria by attaching to receptors on the bacterial cell surface that are often virulence factors, phage-resistant mutants (which lack these receptors) are often less pathogenic than phage-susceptible bacteria (Inal, 2003). Further, the development of phage-resistance can be forestalled altogether if phages are used in cocktails (preparations containing multiple types of phages) and/or in conjunction with antibiotics. In fact, phage therapy and antibiotic therapy, when co-applied, are synergistic (Kutateladze and Adamia, 2010).

Despite the attractions of phage therapy, scientific and logistical challenges remain. Wild-type phage particles are rapidly eliminated by the body's reticuloendothelial (mononuclear phagocyte) system, so in order to enhance phages’ circulatory time and improve the efficacy of treatment, long-circulating mutants (Merril et al., 1996) must be selected, or wild-type virions must be shielded with a non-immunogenic polymer such as polyethylene glycol (Kim et al., 2008). An improved understanding of phages’ *in vivo* pharmacokinetics, including relevant inundation and proliferation thresholds, would also increase phages’ therapeutic value (Cairns et al., 2009). The obstacles to phage therapy are not purely scientific: manufacturing, production, and distribution concerns relating to the scalability of phage therapy have also been discussed (Lu and Koeris, 2011). More broadly, for phage therapy to be useful in clinical settings, a patient's specific etiological agent would need to be rapidly identified and matched to the relevant phage(s) in a comprehensive pre-existing database. Because this scenario is inconsistent with how antibiotics are traditionally employed (Bull et al., 2002), new and interdisciplinary thinking involving bioinformaticists, health care professionals, and phage researchers, among others, would be required to make phage therapy practicable on a large-scale.

Skeptics also argue that phage therapy has not proven its mettle in rigorous, large-scale trials. While it is true that many phage therapy studies did not follow Western protocols, the overall body of evidence suggesting phage therapy's efficacy, when used appropriately, is significant. Phage therapy has been extremely effective at treating a number of bacterial infections in controlled animal studies (Williams Smith et al., 1987; Biswas et al., 2002; Hawkins et al., 2010). The documented successes of phage therapy in Eastern Europe should be taken with a grain of salt, if insufficient by Western standards, but not entirely discounted. Most importantly, large-scale, well-controlled studies attesting to phage therapy's effectiveness do indeed exist. In 1963, health authorities in Tbilisi, Georgia enlisted more than 30,000 children in a blind study and reported that incidences of dysentery were significantly lower amongst those who prophylactically received a weekly phage pill (1.8 cases per 1000) rather than a placebo (6.7 cases per 1000; Sulakvelidze et al., 2001). In a 1983 study involving several hundred patients with suppurative bacterial infections, most of which were drug-resistant, phage therapy achieved a 92.4% overall success rate (Slopek et al., 1987). Most recently, in 2009, a double-blind Phase II clinical study showed phages to be safe and effective at treating chronic drug-resistant ear infections (Wright et al., 2009). These studies and many others (Merril et al., 2003; Kropinksi, 2006; Abedon et al., 2011) hint at a promising future for phage therapy in Western medicine.

Of course, before that future can be fully realized, additional research will be needed, as discussed briefly above. But in order to make that research investment worthwhile, especially (but not exclusively) for the private sector, and in order to ultimately incorporate phage therapy into a larger antibacterial arsenal, a regulatory framework must exist that allows phages to be utilized to their maximum potential.

At its best, phage therapy is a form of personalized medicine because specific phages (usually multiple phages combined as a multivalent cocktail) are carefully selected to treat a patient's specific bacterial infection. Success rates from these customized phages are five- to sixfold higher than that of standardized phage products (Zhukov-Verezhnikov et al., 1978), so the use of personalized phage cocktails has historically been crucial for effective treatment. Nonetheless, the U.S. Food and Drug Administration (FDA), which will decide the fate of phage therapy in the United States and influence its adoption around the world, has not been receptive to this idea. To date, the FDA has essentially grafted its traditional antibiotic regulatory protocols onto phage therapy, meaning that all components of a phage cocktail must go through individual clinical trials and that the composition of these cocktails cannot be altered without re-approval (Thiel, 2004). This policy does not reflect the fundamental differences between phages and antibiotics, and would, if perpetuated, likely render phage therapy both prohibitively expensive and significantly less effective.

Fortunately, there exists at least one regulatory precedent that could be appropriately applied to phage therapy: rather than regulate phage cocktails as it does drug cocktails, the FDA could instead regulate phage cocktails in a manner analogous to the FluMist^®^ influenza vaccine. Each year, FluMist^®^, a live-virus vaccine comprising a cocktail of three or four attenuated influenza strains, is reformulated to most effectively counter circulating flu strains (Marwick, 2000). Rather than mandate separate clinical trials for each season's vaccine, the FDA has instead approved the process by which FluMist^®^ is developed. Such a regulatory model could also be applied to phage cocktails: rather than requiring separate trials for each component of a preparation, the FDA could instead set stringent guidelines on the process by which those cocktails are produced. For instance, the FDA could establish formal standards for the screening of phages (ensuring only lytic phages are used in therapy), the purification of phage preparations (including the removal of endotoxin), and the selection of appropriate phages for patients’ unique infections. If the FDA were to modernize its current stance on phage therapy, scientific certainties, not regulatory uncertainties, could determine the future of this promising treatment.

Phage therapy holds tremendous potential as a powerful way to combat increasingly dangerous bacterial infections. Even as researchers continue to explore the science behind phage therapy, it remains unclear if phage therapy will indeed save lives on a significant scale or if it will ultimately fail to fulfill its promise. One thing seems clear, though: if phage therapy is to move out of the twentieth century and into the twenty-first, so too must the regulatory models that govern it.
